# Outcomes With Pembrolizumab Monotherapy in Patients With Programmed Death-Ligand 1–Positive NSCLC With Brain Metastases: Pooled Analysis of KEYNOTE-001, 010, 024, and 042

**DOI:** 10.1016/j.jtocrr.2021.100205

**Published:** 2021-07-01

**Authors:** Aaron S. Mansfield, Roy S. Herbst, Gilberto de Castro, Rina Hui, Nir Peled, Dong-Wan Kim, Silvia Novello, Miyako Satouchi, Yi-Long Wu, Edward B. Garon, Martin Reck, Andrew G. Robinson, Ayman Samkari, Bilal Piperdi, Victoria Ebiana, Jianxin Lin, Tony S.K. Mok

**Affiliations:** a Division of Medical Oncology, Mayo Clinic, Rochester, Minnesota; b Yale Comprehensive Cancer Center, Yale University School of Medicine, New Haven, Connecticut; c Instituto do Câncer do Estado de São Paulo, São Paulo, Brazil; d Westmead Hospital and the University of Sydney, Sydney, Australia; e Soroka Cancer Center, Ben Gurion University, Beer Sheva, Israel; f Seoul National University Hospital, Seoul National University College of Medicine, Seoul, South Korea; g Azienda Ospedaliero-Universitaria San Luigi Gonzaga, University of Turin, Orbassano, Italy; h Hyogo Cancer Center, Akashi, Japan; i Guangdong Lung Cancer Institute, Guangdong Provincial People’s Hospital and Guangdong Academy of Medical Sciences, Guangdong, China; j David Geffen School of Medicine at the University of California, Los Angeles, Los Angeles, California; k Lung Clinic Grosshansdorf, Airway Research Center North, German Center of Lung Research, Grosshansdorf, Germany; l Cancer Centre of Southeastern Ontario at Kingston General Hospital, Kingston, Ontario, Canada; m Merck & Co., Inc., Kenilworth, New Jersey; n State Key Laboratory of Translational Oncology, Chinese University of Hong Kong, Shatin, Hong Kong Special Administrative Region, China

**Keywords:** Pembrolizumab, Brain metastases, Chemotherapy, Non‒small-cell lung cancer, PD-L1

## Abstract

**Introduction:**

We retrospectively evaluated outcomes in patients with programmed death-ligand 1 (PD-L1)–positive non–small-cell lung cancer (NSCLC) to determine whether baseline (i.e., at study enrollment) brain metastases were associated with the efficacy of pembrolizumab versus chemotherapy.

**Methods:**

We pooled data for patients with previously treated or untreated PD-L1‒positive (tumor proportion score [TPS], ≥1%) advanced or metastatic NSCLC in KEYNOTE-001 (NCT01295827), KEYNOTE-010 (NCT01905657), KEYNOTE-024 (NCT02142738), and KEYNOTE-042 (NCT02220894). Patients received pembrolizumab (2 mg/kg, 10 mg/kg, or 200 mg every 3 wk or 10 mg/kg every 2 wk); chemotherapy was a comparator in all studies except KEYNOTE-001. All studies included patients with previously treated, stable brain metastases.

**Results:**

A total of 3170 patients were included, 293 (9.2%) with and 2877 (90.8%) without baseline brain metastases; median (range) follow-up at data cutoff was 12.9 (0.1‒43.7) months. Pembrolizumab improved overall survival versus chemotherapy in patients with or without baseline brain metastases: benefit was seen in patients with PD-L1 TPS ≥50% (0.67 [95% confidence intervals (CI): 0.44‒1.02] and 0.66 [95% CI: 0.58‒0.76], respectively) and PD-L1 TPS ≥1% (0.83 [95% CI: 0.62‒1.10] and 0.78 [95% CI: 0.71‒0.85], respectively). Progression-free survival was improved, objective response rates were higher, and duration of response was longer with pembrolizumab versus chemotherapy regardless of brain metastasis status. The incidence of treatment-related adverse events with pembrolizumab versus chemotherapy was 66.3% versus 84.4% in patients with brain metastases and 67.2% versus 88.3% in those without.

**Conclusions:**

Pembrolizumab monotherapy improved outcomes and was associated with fewer adverse events than chemotherapy in patients with treatment-naive and previously treated PD-L1‒positive advanced/metastatic NSCLC regardless of the presence of baseline treated, stable brain metastases.

## Introduction

Brain metastases, which occur in approximately one-third of patients with advanced non–small-cell lung cancer (NSCLC) are associated with poorer outcomes compared with other sites of metastasis.^1^ Despite these findings, patients with advanced NSCLC and active brain metastases are often underrepresented in, or excluded from, clinical trials.^2^, ^3^, ^4^, ^5^, ^6^ Recent studies have shown fewer tumor-infiltrating lymphocytes and T-cell clones and less programmed death-ligand 1 (PD-L1) expression in brain metastases than in paired primary lung cancers,^7^^,^^8^ suggesting the potential for differential response to immunotherapy among patients with and without brain metastases. However, recent evidence from patients with NSCLC receiving programmed death 1 (PD-1) or PD-L1 inhibitor monotherapy suggests that the presence of brain metastases was not associated with poorer survival on the basis of multivariate analysis.^9^

The efficacy of monotherapy with the anti–PD-1 monoclonal antibody pembrolizumab has been reported in patients with advanced NSCLC, including a phase 1 study of previously treated and untreated disease (KEYNOTE-001),^10^ a phase 2/3 study (KEYNOTE-010) in the second-line or later setting,^11^ and two phase 3 studies in the first-line setting (KEYNOTE-024 and -042).^12^^,^^13^ In the three randomized studies (KEYNOTE-010, -024, and -042), overall survival (OS) was significantly longer with pembrolizumab than chemotherapy. Each of the four studies permitted enrollment of patients with previously treated brain metastases provided the patients were clinically stable.

We sought to better characterize outcomes in this population of patients with historically poor prognoses. In this pooled analysis, we retrospectively evaluated outcomes in patients with PD-L1–positive NSCLC, with or without known baseline brain metastases, who were treated with pembrolizumab monotherapy in KEYNOTE-001 and either pembrolizumab monotherapy or chemotherapy in KEYNOTE-010, -024, and -042.

## Materials and Methods

### Patients

Patients from KEYNOTE-001 (NCT01295827),^10^ KEYNOTE-010 (NCT01905657),^11^ KEYNOTE-024 (NCT02142738),^12^ and KEYNOTE-042 (NCT02220894)^13^ were included in this post hoc pooled analysis. Methods for each study were described previously and are briefly summarized here. The study protocols were approved by institutional review boards or ethics committees at each site. Patients provided written informed consent.

In all studies, eligible patients were at least 18 years of age with an Eastern Cooperative Oncology Group (ECOG) performance status of 0 or 1, adequate organ function, life expectancy of at least 3 months, a histologically or cytologically confirmed diagnosis of NSCLC, and at least 1 measurable lesion per Response Evaluation Criteria in Solid Tumors version 1.1 (RECIST v1.1).^14^ In KEYNOTE-001, patients had previously treated or untreated locally advanced or metastatic NSCLC and could have had any PD-L1 tumor proportion score (TPS), although, for this pooled analysis, only patients with PD-L1–positive tumors (i.e., PD-L1 TPS ≥1%) were included. In KEYNOTE-010, patients had previously treated advanced NSCLC and PD-L1 TPS ≥1%. Both KEYNOTE-001 and -010 allowed *EGFR*-mutant or *ALK*-translocated tumors that had failed tyrosine kinase inhibitor therapy. In KEYNOTE-024, patients had treatment-naive advanced NSCLC with no sensitizing *EGFR* or *ALK* genomic tumor aberrations and PD-L1 TPS ≥50%. In KEYNOTE-042, patients had treatment-naive advanced NSCLC with no sensitizing *EGFR* or *ALK* genomic tumor aberrations and PD-L1 TPS ≥1%. Patients with known active central nervous system (CNS) metastases and/or carcinomatous meningitis were excluded from each study. Patients with previously treated brain metastases were eligible provided they were clinically stable for at least 4 weeks before study entry, showed no evidence of new or enlarging brain metastases, and completed corticosteroid treatment for brain metastases greater than or equal to 3 days (KEYNOTE-010, -024, -042) or at least 7 days (KEYNOTE-001) before study treatment.

### Study Design and Treatment

Patients in KEYNOTE-001 received intravenous pembrolizumab 2 mg/kg or 10 mg/kg every 3 weeks or 10 mg/kg every 2 weeks. Patients in KEYNOTE-010 were randomized 1:1:1 to receive intravenous pembrolizumab 2 mg/kg, 10 mg/kg, or docetaxel 75 mg/m^2^ every 3 weeks for up to 35 cycles; randomization was stratified by ECOG performance status (0 versus 1), region (East Asia versus non–East Asia), and PD-L1 TPS (≥50% versus 1%–49%). Patients in KEYNOTE-024 were randomized 1-to-1 to receive intravenous pembrolizumab 200 mg every 3 weeks for up to 35 cycles or platinum-based chemotherapy for four to six cycles with optional pemetrexed maintenance for nonsquamous histologies; randomization was stratified by histology (squamous versus nonsquamous), ECOG performance status (0 versus 1), and region (East Asia versus non–East Asia). Patients in KEYNOTE-042 were randomized 1-to-1 to receive intravenous pembrolizumab 200 mg every 3 weeks for up to 35 cycles or platinum-based chemotherapy for four to six cycles with optional pemetrexed maintenance for nonsquamous histologies; randomization was stratified by ECOG performance status (0 versus 1), histology (squamous versus nonsquamous), region (East Asia versus non–East Asia), and PD-L1 TPS (≥50% versus 1%–49%).

### Assessments

Radiographic imaging with computed tomography or magnetic resonance imaging (MRI) for non-CNS assessment was done within 30 days of enrollment (baseline) and every 9 weeks thereafter. For KEYNOTE-042, imaging was performed every 9 weeks for the first 45 weeks and then every 12 weeks thereafter. In all studies, the response was assessed per RECIST v1.1 by blinded, independent central review.^14^ All patients in KEYNOTE-024 had CNS imaging at screening, including patients with no previous history of brain metastases; in the other studies, patients with previously treated brain metastases had to exhibit no evidence of new or enlarging brain metastases for at least 4 weeks after treatment of the brain metastases. In all studies, regular CNS imaging was not required at subsequent imaging assessments. Adverse events (AEs) were monitored during and for 30 days after treatment and graded per National Cancer Institute Common Terminology Criteria for Adverse Events version 4.0. Serious AEs were monitored for 90 days after treatment. PD-L1 expression status was determined by a central laboratory in formalin-fixed tumor samples collected at the time of metastatic disease diagnosis. All studies used a 22C3 antibody‒based assay to evaluate PD-L1 TPS.

### End points

End points evaluated were OS, progression-free survival (PFS), objective response rate (ORR), duration of response (DOR), and incidence of AEs. OS was defined as the time from randomization (first dose of study treatment in KEYNOTE-001) to death from any cause. PFS was defined as the time from randomization (first dose of study treatment in KEYNOTE-001) to progression, defined by RECIST v1.1, or death from any cause. ORR was defined as the proportion of patients with radiologically confirmed complete or partial responses. DOR, determined for patients with a complete or partial response, was defined as the time from first documented evidence of response until disease progression.

### Statistical Analysis

This pooled analysis included individual patient data from patients with PD-L1 TPS ≥1% enrolled in KEYNOTE-001 and all patients from the intent-to-treat populations enrolled in KEYNOTE-010, -024, and -042. One patient in KEYNOTE-010 was excluded from efficacy analyses because their prebaseline scans were not compliant with the protocol, preventing an adequate assessment of tumor response. Efficacy was evaluated in the pooled intent-to-treat population (PD-L1 TPS ≥1%) and PD-L1 TPS ≥50% population; safety was evaluated in the pooled population of patients who received at least one dose of study treatment. All analyses were descriptive and not controlled for multiplicity.

The Kaplan-Meier method was used to estimate OS, PFS, and DOR. For OS and PFS, hazard ratios (HRs) and 95% confidence intervals (CIs) of the treatment differences were based on the Cox proportional hazards regression model with treatment as a covariate.

## Results

### Patient Disposition

**Figure 1:**
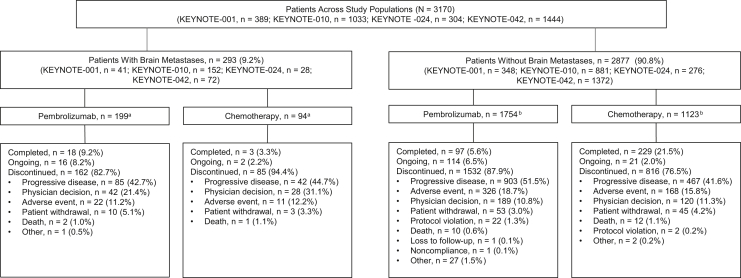
Patient disposition of pooled analysis. We included patients from the KEYNOTE-042 China extension study in this analysis; therefore, the number of enrolled patients is higher than previously reported.^13^^a^Three patients allocated to pembrolizumab and four to chemotherapy did not receive study treatment. ^b^A total of 11 patients allocated to pembrolizumab and 57 to chemotherapy did not receive study treatment.

Of the 3170 patients included in this pooled analysis, 293 (9.2%) had baseline brain metastases and 2877 (90.8%) had no known baseline brain metastases. Of the patients with brain metastases, 199 (67.9%) were assigned to pembrolizumab and 94 (32.1%) to chemotherapy. Of the patients without known brain metastases, 1754 (61.0%) and 1123 patients (39.0%) were assigned to pembrolizumab and chemotherapy, respectively (Fig. 1). Data cutoff dates were November 5, 2018 (KEYNOTE-001), March 16, 2018 (KEYNOTE-010), July 10, 2017 (KEYNOTE-024), and September 4, 2018 (KEYNOTE-042). Median (range) duration of follow-up was 12.9 (0.1−43.7) months overall, 18.4 (0.5–43.7) for patients with brain metastases, and 12.6 (0.1–42.5) months for patients without brain metastases.

Baseline characteristics were generally similar between patients with and without brain metastases (Table 1), although the brain metastases group had a lower percentage of men (51.9% versus 66.9%), a higher percentage of patients with nonsquamous histology (86.0% versus 65.7%) and *EGFR* (11.3% versus 3.6%) genetic aberrations, and a lower percentage of treatment-naive patients (34.5% versus 59.4%). Approximately half of the patients with (54.6%) and without (50.1%) brain metastases had PD-L1 TPS ≥50% at baseline.

**Table 1:** Baseline Characteristics in Patients With and Without Brain Metastases (Pooled Intent-to-Treat Population)

Characteristics	With Brain Metastases		Without Brain Metastases	
	Pembrolizumab (n = 199)	Chemotherapy (n = 94)	Pembrolizumab (n = 1754)	Chemotherapy (n = 1123)
Male	99 (49.7)	53 (56.4)	1146 (65.3)	778 (69.3)
Age, median (range), y	59.0 (31–88)	60.0 (31–81)	64.0 (20–93)	64.0 (32–90)
ECOG				
0	59 (29.6)	29 (30.9)	580 (33.1)	348 (31.0)
1	139 (69.8)	65 (69.1)	1167 (66.5)	772 (68.7)
2 or 3^a^	1 (0.5)	0	5 (0.3)	2 (0.2)
Unknown or missing	0	0	2 (0.1)	1 (0.1)
Smoking history				
Current or former	159 (79.9)	77 (81.9)	1407 (80.2)	888 (79.1)
Never	39 (19.6)	15 (16.0)	346 (19.7)	230 (20.5)
Missing	1 (0.5)	2 (2.1)	1 (0.1)	5 (0.4)
Histology				
Nonsquamous	173 (86.9)	79 (84.0)	1181 (67.3)	709 (63.1)
Squamous	21 (10.6)	8 (8.5)	523 (29.8)	384 (34.2)
Other or unknown	5 (2.5)	7 (7.4)	50 (2.9)	30 (2.7)
*EGFR* mutation^b^	27 (13.6)	6 (6.4)	82 (4.7)	21 (1.9)
*ALK* translocation^b^	2 (1.0)	0	13 (0.7)	2 (0.2)
Previous systemic therapies^c^				
0	55 (27.6)	46 (48.9)	886 (50.5)	822 (73.2)
1	61 (30.7)	29 (30.9)	509 (29.0)	215 (19.1)
≥2	83 (41.7)	19 (20.2)	359 (20.5)	86 (7.7)
PD-L1 TPS				
≥50%	112 (56.3)	48 (51.1)	842 (48.0)	598 (53.3)
1%–49%	87 (43.7)	46 (48.9)	912 (52.0)	525 (46.7)

*Note:* Values are n (%) of patients unless indicated otherwise.

ECOG, Eastern Cooperative Oncology Group; PD-L1, programmed death-ligand-1; TPS, tumor proportion score.

a Most patients with an ECOG performance status of 2 or 3 during screening improved to 1 by the time the patients were randomized.

b Patients with *EGFR* or *ALK* genomic tumor aberrations were not excluded from enrollment in KEYNOTE-001 or KEYNOTE-010.

c Includes adjuvant and neoadjuvant therapies.

### Overall Survival

At data cutoff, among patients with baseline brain metastases, 139 of 199 patients (69.8%) in the pembrolizumab group and 70 of 94 (74.5%) in the chemotherapy group had died. Among patients without baseline brain metastases, 1245 of 1754 patients (71.0%) in the pembrolizumab group and 846 of 1123 (75.3%) in the chemotherapy group had died.

**Figure 2:**
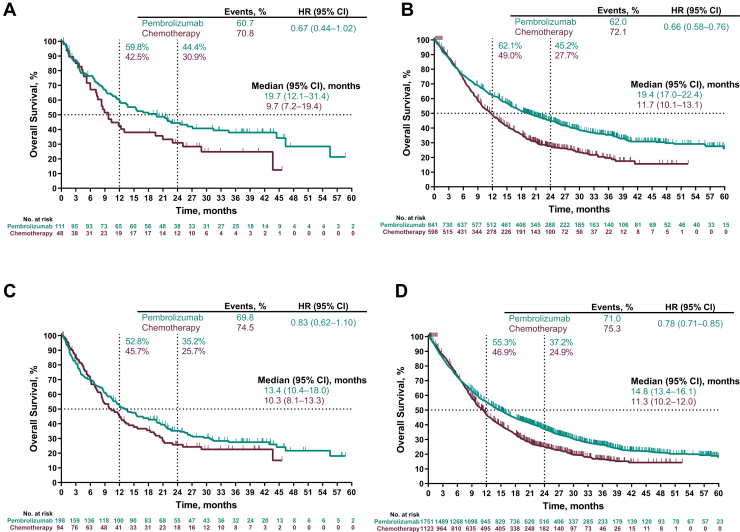
Overall survival in patients with a PD-L1 TPS of ≥50% in patients *(A)* with and *(B)* without baseline brain metastases and in patients with a PD-L1 TPS of ≥1% in patients *(C)* with and *(D)* without baseline brain metastases. Four patients in the pooled intent-to-treat population had missing overall survival data. CI, confidence interval; HR, hazard ratio; PD-L1, programmed death-ligand 1; TPS, tumor proportion score.

Among patients with PD-L1 TPS ≥50% with brain metastases at baseline, the HR for OS (pembrolizumab versus chemotherapy) was 0.67 (95% CI: 0.44–1.02); median OS was 19.7 (95% CI: 12.1–31.4) and 9.7 (95% CI: 7.2–19.4) months, respectively (Fig. 2*A*). Among patients with PD-L1 TPS ≥50% without brain metastases, the HR for OS was 0.66 (95% CI: 0.58–0.76); median OS was 19.4 (95% CI: 17.0–22.4) and 11.7 (95% CI: 10.1–13.1) months, respectively (Fig. 2*B*).

OS also favored the pembrolizumab group among patients with PD-L1 TPS ≥1%. Among patients with brain metastases, the HR for OS was 0.83 (95% CI: 0.62–1.10); median OS was 13.4 (95% CI: 10.4–18.0) and 10.3 (95% CI: 8.1–13.3) months, respectively (Fig. 2*C*). For patients without brain metastases, the HR for OS was 0.78 (95% CI: 0.71–0.85); median OS was 14.8 (95% CI: 13.4–16.1) and 11.3 (95% CI: 10.2–12.0) months, respectively (Fig. 2*D*).

### Progression-Free Survival

**Figure 3:**
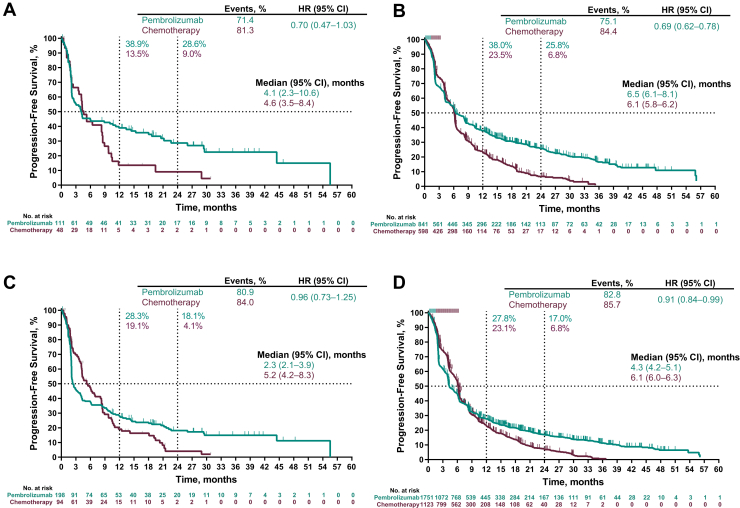
Progression-free survival in patients with a PD-L1 TPS of ≥50% in patients *(A)* with and *(B)* without baseline brain metastases and in patients with a PD-L1 TPS of ≥1% in patients *(C)* with and *(D)* without baseline brain metastases. The response was assessed per Response Evaluation Criteria in Solid Tumors version 1.1 by blinded, independent central review. Four patients in the pooled intent-to-treat population had missing progression-free survival data. CI, confidence interval; HR, hazard ratio; PD-L1, programmed death-ligand 1; TPS, tumor proportion score.

At data cutoff, among patients with baseline brain metastases, 161 of 199 patients (80.9%) in the pembrolizumab group and 79 of 94 (84.0%) in the chemotherapy group had disease progression or died. Among patients without baseline brain metastases, 1452 of 1754 patients (82.8%) in the pembrolizumab group and 962 of 1123 (85.7%) in the chemotherapy group had disease progression or died. Among patients with PD-L1 TPS ≥50%, the HR for PFS (pembrolizumab versus chemotherapy) was 0.70 (95% CI: 0.47–1.03) in patients with baseline brain metastases (Fig. 3*A*) and 0.69 (95% CI: 0.62–0.78) in patients without (Fig. 3*B*). Among patients with PD-L1 TPS ≥1%, the HR for PFS was 0.96 (95% CI: 0.73–1.25) in patients with brain metastases (Fig. 3*C*) and 0.91 (95% CI: 0.84–0.99) in patients without (Fig. 3*D*).

### Systemic ORR

ORR was higher among patients assigned to pembrolizumab versus chemotherapy irrespective of baseline brain metastasis status (Table 2). Among patients with PD-L1 TPS ≥50%, ORR with pembrolizumab versus chemotherapy was 33.9% versus 14.6% in patients with brain metastases and 38.1% versus 26.1% in patients without. Among patients with PD-L1 TPS ≥1%, ORR with pembrolizumab versus chemotherapy was 26.1% versus 18.1% in patients with brain metastases and 25.8% versus 22.2% in patients without.

**Table 2:** ORR in Patients With and Without Brain Metastases by PD-L1 TPS (Pooled Intent-to-Treat Population)

Outcome	With Brain Metastases		Without Brain Metastases	
	Pembrolizumab	Chemotherapy	Pembrolizumab	Chemotherapy
PD-L1 TPS, ≥50%, n	112	48	842	598
ORR, n (%) [95% CI]	38 (33.9) [25.3–43.5]	7 (14.6) [6.1–27.8]	321 (38.1) [34.8–41.5]	156 (26.1) [22.6–29.8]
Response, n (%)				
Complete response	3 (2.7)	0	22 (2.6)	2 (0.3)
Partial response	35 (31.3)	7 (14.6)	299 (35.5)	154 (25.8)
Stable disease	24 (21.4)	19 (39.6)	233 (27.7)	246 (41.1)
Progressive disease	36 (32.1)	11 (22.9)	190 (22.6)	97 (16.2)
Not evaluable	3 (2.7)	2 (4.2)	18 (2.1)	11 (1.8)
No assessment	9 (8.0)	9 (18.8)	75 (8.9)	88 (14.7)
Response duration, median (range), mo	NR (4.0+ to 41.7+)	7.6 (2.9+ to 28.6+)	33.9 (1.4+ to 49.3+)	8.2 (1.6+ to 30.4+)
PD-L1 TPS ≥1%, n	199	94	1754	1123
ORR, n (%) [95% CI]	52 (26.1) [20.2–32.8]	17 (18.1) [10.9–27.4]	452 (25.8) [23.7–27.9]	249 (22.2) [19.8–24.7]
Response, n (%)				
Complete response	3 (1.5)	1 (1.1)	29 (1.7)	3 (0.3)
Partial response	49 (24.6)	16 (17.0)	423 (24.1)	246 (21.9)
Stable disease	37 (18.6)	41 (43.6)	604 (34.4)	517 (46.0)
Progressive disease	80 (40.2)	18 (19.1)	474 (27.0)	174 (15.5)
Not evaluable	7 (3.5)	4 (4.3)	38 (2.2)	23 (2.0)
No assessment	21 (10.6)	14 (14.9)	169 (9.6)	160 (14.2)
Response duration, median (range), mo	NR (3.3 to 46.2+)	8.3 (2.0+ to 28.6+)	30.4 (1.4+ to 49.3+)	8.1 (1.1+ to 30.4+)

*Note:* Responses were based on blinded, independent central review assessment per RECIST version 1.1.

CI, confidence interval; NR, not reached; ORR, objective response rate; PD-L1, programmed death ligand-1; RECIST, Response Evaluation Criteria in Solid Tumors; TPS, tumor proportion score.

DOR was longer among patients who received pembrolizumab. Among patients with PD-L1 TPS ≥50%, median (range) DOR was not reached (4.0+ to 41.7+ mo) in the pembrolizumab group and was 7.6 (2.9+ to 28.6+) months in the chemotherapy group among patients with brain metastases and was 33.9 (1.4+ to 49.3+) and 8.2 (1.6+ to 30.4+) months, respectively, among patients without brain metastases (+ indicates no progressive disease at the time of last disease assessment). Among patients with PD-L1 TPS ≥1%, median (range) DOR was not reached (3.3 to 46.2+ mo) in the pembrolizumab group and was 8.3 (2.0+ to 28.6+) months in the chemotherapy group among patients with brain metastases and was 30.4 (1.4+ to 49.3+) and 8.1 (1.1+ to 30.4+) months, respectively, among patients without.

### Safety

The median (range) treatment duration for patients with baseline brain metastases given pembrolizumab or chemotherapy was 2.8 (0.03–39.6) and 2.9 (0.03–29.5) months, respectively. In this population, treatment-related AEs of any grade occurred in 130 of 196 patients (66.3%) in the pembrolizumab group and 76 of 90 (84.4%) in the chemotherapy group (Table 3). Treatment-related AEs of greater than or equal to grade 3 occurred in 29 patients (14.8%) and 41 patients (45.6%), respectively. Treatment-related AEs resulted in the discontinuation of study treatment for 12 patients (6.1%) in the pembrolizumab group and 9 (10.0%) in the chemotherapy group; three (1.5%) and three patients (3.3%), respectively, died of treatment-related AEs. A total of 19 patients (9.7%) had at least one treatment-related AE affecting the nervous system in the pembrolizumab group compared with 24 (26.7%) in the chemotherapy group; the most common are detailed in Table 3. Immune-mediated AEs and infusion reactions, regardless of relationship to study treatment, occurred in 41 patients (20.9%) and eight patients (8.9%) in the pembrolizumab and chemotherapy groups, respectively.

**Table 3:** Treatment-Related AEs in Patients With and Without Brain Metastases (Pooled Safety Population)

Treatment-Related AEs	With Brain Metastases		Without Brain Metastases	
	Pembrolizumab (n = 196)	Chemotherapy (n = 90)	Pembrolizumab (n = 1743)	Chemotherapy (n = 1066)
Any	130 (66.3)	76 (84.4)	1172 (67.2)	941 (88.3)
Grade ≥3	29 (14.8)	41 (45.6)	311 (17.8)	460 (43.2)
Led to discontinuation of study treatment	12 (6.1)	9 (10.0)	144 (8.3)	117 (11.0)
Led to death	3 (1.5)	3 (3.3)	22 (1.3)	21 (2.0)
Affected the nervous system	19 (9.7)	24 (26.7)	122 (7.0)	283 (26.5)
Most common (≥2% in any group)				
Neuropathy peripheral	1 (0.5)	7 (7.8)	9 (0.5)	83 (7.8)
Dysgeusia	3 (1.5)	8 (8.9)	23 (1.3)	45 (4.2)
Peripheral sensory neuropathy	1 (0.5)	3 (3.3)	12 (0.7)	58 (5.4)
Paresthesia	1 (0.5)	5 (5.6)	12 (0.7)	34 (3.2)
Headache	7 (3.6)	3 (3.3)	24 (1.4)	11 (1.0)
Hypesthesia	0	1 (1.1)	3 (0.2)	25 (2.3)
Immune-mediated AEs and infusion reactions^a^	41 (20.9)	8 (8.9)	440 (25.2)	80 (7.5)
Grade 3−5	10 (5.1)	1 (1.1)	129 (7.4)	17 (1.6)

*Note:* AEs were graded on the basis of National Cancer Institute Common Terminology Criteria for Adverse Events, version 4.03. Values are n (%) of patients.

AEs, adverse events.

a Immune-mediated AEs were classified on the basis of a list of preferred terms identified by the sponsor as having an immune etiology. All immune-mediated AEs and infusion reactions are included, regardless of relationship to study drug.

Safety results in patients without brain metastases (Table 3) were consistent with those in patients with brain metastases. The median (range) treatment duration for patients without brain metastases given pembrolizumab and chemotherapy was 4.7 (0.03–76.0) and 3.5 (0.03–34.8) months, respectively.

## Discussion

In this pooled analysis, pembrolizumab monotherapy improved clinical outcomes versus chemotherapy in patients with PD-L1–positive advanced or metastatic NSCLC, irrespective of the presence of treated, stable brain metastases at baseline. The OS and PFS benefits of pembrolizumab in patients with brain metastases were similar to that reported in patients without known brain metastases. ORRs were higher with pembrolizumab than with chemotherapy, and median DOR was not reached in the pembrolizumab group. Consistent with the individual study results, this pooled analysis showed a greater magnitude of benefit in patients with PD-L1 TPS ≥50% versus PD-L1 TPS ≥1%. Pembrolizumab had a manageable safety profile both in patients with and without baseline brain metastases.

A phase 2 study provided evidence that pembrolizumab had activity in the CNS comparable with that in systemic disease.^15^^,^^16^ The updated analysis included 42 patients with NSCLC and untreated or progressive brain metastases on the basis of brain MRI.^16^ The brain metastasis response rate (primary end point) was 30% in the PD-L1−positive NSCLC cohort with measurable disease, with no responses in the PD-L1−negative cohort. CNS responses were durable (DOR = 5.7 mo), with 10 of 11 patients still responding at the time of the last on-study MRI.^16^ The mechanism by which immunotherapy might be effective within the CNS is an area of ongoing investigation. Brain metastases disrupt the integrity of the blood-brain barrier and blood-cerebrospinal fluid barrier, with several studies now reporting the presence of tumor-infiltrating lymphocytes in brain metastases of several primary cancers, including NSCLC.^17^

Our results are consistent with those of previous trials in which monotherapy with other PD-1 and PD-L1 inhibitors has shown benefit in patients with advanced NSCLC with and without brain metastases,^18^^,^^19^ lending further support of a role for immunotherapy in these patients. Our pooled population comprised a large sample size, but the proportions of patients with treated and stable brain metastases were similar in our analysis (10.2%) and the previous studies (9.5%−14.5%).^18^^,^^19^ In our analysis, median OS was 19.7 and 13.4 months in patients receiving pembrolizumab with brain metastases and PD-L1 TPS ≥50% and PD-L1 TPS ≥1%, respectively, which was greater than the median OS in the chemotherapy arm (9.7 and 10.3 mo, respectively); HRs for OS were 0.67 (95% CI: 0.44‒1.02) and 0.83 (95% CI: 0.62‒1.10), respectively. A similar pattern was observed in the phase 3 study of atezolizumab in which median OS was 16.0 months with atezolizumab versus 11.9 months with docetaxel. Real-world evidence in patients with advanced NSCLC treated with first-line or later pembrolizumab, with or without chemotherapy, or second-line or later nivolumab parallels the clinical trial experience reporting similar benefits in patients presenting with and without brain metastases.^20^, ^21^, ^22^

No new safety signals were identified in our analysis. The safety profile of pembrolizumab monotherapy was similar in patients with and without baseline brain metastases and was more favorable than that of chemotherapy. The incidences of treatment-related AEs, treatment-related AEs of grade ≥3, and those leading to death, discontinuation of study treatment, or affecting the nervous system were all lower with pembrolizumab versus chemotherapy. The presence of brain metastases was not associated with an increased incidence of nervous system events in either treatment group. Our results are generally consistent with those of other PD-1 and PD-L1 inhibitors.^19^^,^^22^ In contrast to our findings with pembrolizumab, one randomized controlled trial reported less-favorable safety in a relatively small sample of patients with advanced NSCLC with brain metastases (n = 60) compared with those without brain metastases (n = 362).^18^ In that trial, among patients treated with atezolizumab, those with brain metastases experienced more treatment-related AEs, including events that were grade ≥3, serious, or neurologic.^18^

Our results parallel those of first-line combination therapy with pembrolizumab plus platinum-based chemotherapy versus platinum-based chemotherapy alone in patients with advanced NSCLC with and without baseline brain metastases.^23^ In that analysis, which pooled results from three clinical trials (KEYNOTE-021, -189, and -407), combination therapy was associated with improved outcomes, regardless of the presence of brain metastases, and a manageable safety profile.

A strength of our analysis is that we evaluated outcomes by pooling data across four individual trials, allowing for a robust assessment in this clinically important group. Limitations include the fact that no adjustments were made for multiplicity and outcomes were retrospectively evaluated. However, exploratory subgroup analyses among patients with brain metastases were prespecified in all studies. Another limitation is that patients with untreated or active brain metastases were not enrolled in any of the trials. Moreover, CNS imaging was not uniformly collected at every individual time point for response assessment in any of the studies, and intracranial responses were not specifically collected; the central reader was not asked to evaluate intracranial responses nor specify the site of progressive disease. Thus, whether there was any benefit of pembrolizumab on intracranial response, intracranial disease progression, and brain-related DOR and PFS could not be assessed. Details on prior local therapy for brain metastases were not captured appropriately, and hence, not analyzed. Characteristics of the studies pooled may also have influenced the outcome. For example, KEYNOTE-001 enrolled predominantly previously treated patients and lacked a control arm. Thus, in the pooled analysis, the chemotherapy arm had a greater percentage of treatment-naive patients, which may have introduced bias in favor of chemotherapy for end points such as OS. Despite this potential bias, the between-treatment group differences in OS favored pembrolizumab. In addition, KEYNOTE-024 included only patients with PD-L1 TPS ≥50%, and thus the PD-L1 TPS ≥1% group in our analysis may be enriched for PD-L1 TPS ≥50% patients. However, given the relatively small number of patients in KEYNOTE-024 (N = 305) compared with the total population in this pooled analysis, this is unlikely to have a substantial impact on the overall findings.

In conclusion, pembrolizumab monotherapy improved clinical outcomes with fewer AEs than chemotherapy in patients with treatment-naive and previously treated PD-L1‒positive, advanced or metastatic NSCLC, including those with treated, stable brain metastases. Clinical outcomes associated with pembrolizumab were similar in patients with and without brain metastases. Pembrolizumab monotherapy is a standard-of-care therapy in the population studied.

## CRediT Authorship Contribution Statement

**Aaron S. Mansfield:** Conceptualization, Methodology, Investigation, Formal analysis, Writing–original draft, Writing–review and editing.

**Roy S. Herbst:** Conceptualization, Methodology, Investigation, Formal analysis, Writing–review and editing.

**Gilberto de Castro Jr., Rina Hui, Martin Reck:** Investigation, Formal Analysis, Writing–review and editing, Resources.

**Nir Peled, Silvia Novello, Andrew G. Robinson, Ayman Samkari:** Investigation, Writing–review and editing.

**Dong-Wan Kim:** Investigation, Writing–review and editing, Resources

**Miyako Satouchi, Yi-Long Wu, Edward B. Garon, Victoria Ebiana:** Investigation, Formal analysis, Writing–review and editing.

**Bilal Piperdi:** Conceptualization, Methodology, Formal Analysis, Data Curation, Writing–original draft, Writing–review and editing, Visualization, Supervision, Project Administration, Funding Acquisition.

**Jianxin Lin:** Software, Validation, Formal Analysis, Data Curation, Writing–review and editing, Visualization, Funding Acquisition.

**Tony S. K. Mok**: Conceptualization, Methodology, Formal analysis, Investigation, Writing–original draft, Writing–review and editing, Resources.

## Data Sharing Statement

Merck Sharp & Dohme Corp., a subsidiary of Merck & Co., Inc., Kenilworth, New Jersey (Merck Sharp & Dohme) is committed to providing qualified scientific researchers access to anonymized data and clinical study reports from the company’s clinical trials for the purpose of conducting legitimate scientific research. Merck Sharp & Dohme is also obligated to protect the rights and privacy of trial participants and, as such, has a procedure in place for evaluating and fulfilling requests for sharing company clinical trial data with qualified external scientific researchers. The Merck Sharp & Dohme data sharing website (available at: http://engagezone.msd.com/ds_documentation.php) outlines the process and requirements for submitting a data request. Applications will be promptly assessed for completeness and policy compliance. Feasible requests will be reviewed by a committee of Merck Sharp & Dohme subject matter experts to assess the scientific validity of the request and the qualifications of the requestors. In line with data privacy legislation, submitters of approved requests must enter into a standard data sharing agreement with Merck Sharp & Dohme before data access is granted. Data will be made available for request after product approval in the United States and European Union or after product development is discontinued. There are circumstances that may prevent Merck Sharp & Dohme from sharing requested data, including country or region-specific regulations. If the request is declined, it will be communicated to the investigator. Access to genetic or exploratory biomarker data requires a detailed, hypothesis-driven statistical analysis plan that is collaboratively developed by the requestor and Merck Sharp & Dohme subject matter experts; after approval of the statistical analysis plan and execution of a data-sharing agreement, Merck Sharp & Dohme will either perform the proposed analyses and share the results with the requestor or will construct biomarker covariates and add them to a file with clinical data that is uploaded to an analysis portal so that the requestor can perform the proposed analyses.
